# The Application of High-Throughput Technologies for the Study of Microbiome and Cancer

**DOI:** 10.3389/fgene.2021.699793

**Published:** 2021-07-28

**Authors:** Lu Qi Wei, Io Hong Cheong, Guang Huan Yang, Xiao Guang Li, Zisis Kozlakidis, Lei Ding, Ning Ning Liu, Hui Wang

**Affiliations:** ^1^State Key Laboratory of Oncogenes and Related Genes, Centre for Single-Cell Omics, School of Public Health, Shanghai Jiao Tong University School of Medicine, Shanghai, China; ^2^International Agency for Research on Cancer, World Health Organization, Lyon, France

**Keywords:** microbiome, high-throughput, cancer, microbiome diversity, functional analysis, cancer therapy

## Abstract

Human gut microbiome research, especially gut microbiome, has been developing at a considerable pace over the last decades, driven by a rapid technological advancement. The emergence of high-throughput technologies, such as genomics, transcriptomics, and others, has afforded the generation of large volumes of data, and in relation to specific pathologies such as different cancer types. The current review identifies high-throughput technologies as they have been implemented in the study of microbiome and cancer. Four main thematic areas have emerged: the characterization of microbial diversity and composition, microbial functional analyses, biomarker prediction, and, lastly, potential therapeutic applications. The majority of studies identified focus on the microbiome diversity characterization, which is reaching technological maturity, while the remaining three thematic areas could be described as emerging.

## Introduction

Human microbiome research has been developing at a considerable pace over the last two decades, partly driven by technological advancement and the ability for high-throughput, culture-independent analyses, and in part because the ability to analyze and interpret the increasing quantities of data has now become possible. As in any rapidly evolving field, there can emerge differences in the definition. For the purposes of this study, which focuses on the human microbiome, especially gut microbiome, the term microbiome aligns with previously reported ones and refers to the entire habitat view, including the microorganisms, their genomes, and associated clinical metadata (Marchesi and Ravel, 2015).

The human microbiome is a dynamic collection of bacteria, viruses, and fungi. Under ideal conditions, these organisms live symbiotically with their human host in gut (Lynch and Pedersen, 2016), and individual species and/or collective bacterial functions under certain conditions may confer many benefits throughout their host's life by metabolizing dietary compounds, educating the immune system, defending against pathogens, and contributing to overall health (Kau et al., 2011; Sharon et al., 2016; Valdes et al., 2018). Therefore, it is critical to try and understand the microbiome as it impacts on a multitude of aspects, including a wide range of pathologies. Accordingly, numerous avenues of research are being pursued to understand what constitutes healthy and abnormal microbiomes (Schwartz et al., 2020), and how they relate to specific disease conditions, such as cancer.

In terms of the latter, historically the first close link between cancer research and the microbiome was achieved already a few decades ago. Specifically, *Helicobacter pylori* was first identified in the late 1970s by J. Robin Warren in gastric tissue samples from patients with chronic gastritis, which was an inflammatory precursor of gastric cancer (Warren and Marshall, 1983). Wotherspoon et al. found 101 (92%) *H. pylori* infection cases out of 110 cases of gastric mucosa-associated lymphoma using modified Giemsa or cresyl violet stain (Wotherspoon et al., 1991). Additionally, the association of *H. pylori* infection with the risk of gastric carcinoma was confirmed in a nested case-control study in 1991 by ELISA assay (Parsonnet et al., 1991). From the history of investigation into this relationship of *H. pylori* infection and chronic gastritis, leading to gastric cancer, the field moved into more extensive studies on the microbiome and its relationship with cancer.

The earliest microbial diversity detection was carried out through microscopic observation (Van Leewenhoeck, 1677) and established microbial isolation and culture technologies (Janssen et al., 2002; Kaeberlein et al., 2002). However, although pure-culture technologies were improved significantly (Browne et al., 2016), the overall knowledge and view of microbial diversity were still limited due to the natural difficulties of laboratory cultivation (Amann et al., 1995; Fredricks et al., 2005). Therefore, as an additional means to the morphological observation and selection of growth conditions, microbiologists also took advantage of the metabolic properties to distinguish different microbes (Pace, 1997). The Biolog technology was successfully developed by BIOLOG in 1989 for carrying out the biochemical reaction test of 95 unique carbon sources and was initially applied to the identification of pure microorganisms (Garland and Mills, 1991).

Beyond these early historical examples, the current era of laboratory automation has ushered-omics technologies, which are increasingly high-throughput, allowing for the detailed characterization of collected samples and specimens from patients and healthy individuals alike. Still, the efforts are mostly concentrating on the accurate characterization of the diversity of the microbiome (and its progressive changes over time in the case of sequential sampling), leading to the interpretation of these observations. While interventions have started taking place (DeFilipp et al., 2018; Smibert et al., 2019; Wing and Kremenchutzky, 2019), these are at the initial stages and not yet an established clinical practice. It is anticipated that the increased understanding in this field through high-throughput laboratory methodologies will lead to future interventions, as well as preventive actions in relation to cancer development. Previous reviews have summarized high-throughput sequencing technologies and the platforms used (Reuter et al., 2015), reviewed shotgun metagenomics process in detail (Quince et al., 2017) or its application in microbiome and several diseases (Wang and Jia, 2016), discussed the gut microbiome (virome) in health or disease situation (Carding et al., 2017), or reviewed investigations on microbiome and cancer (Contreras et al., 2016; Helmink et al., 2019). Notwithstanding the above, the current manuscript is a systematic review on the subject of the high-throughput methodologies that have been employed over the last two decades in the study of the human gut microbiome in relation to cancer. It provides a useful benchmark on current technological developments, biological interpretations, and how the latter might eventually influence clinical practice.

## Methods

### Data Sources and Literature Search Strategy

**Figure 1:**
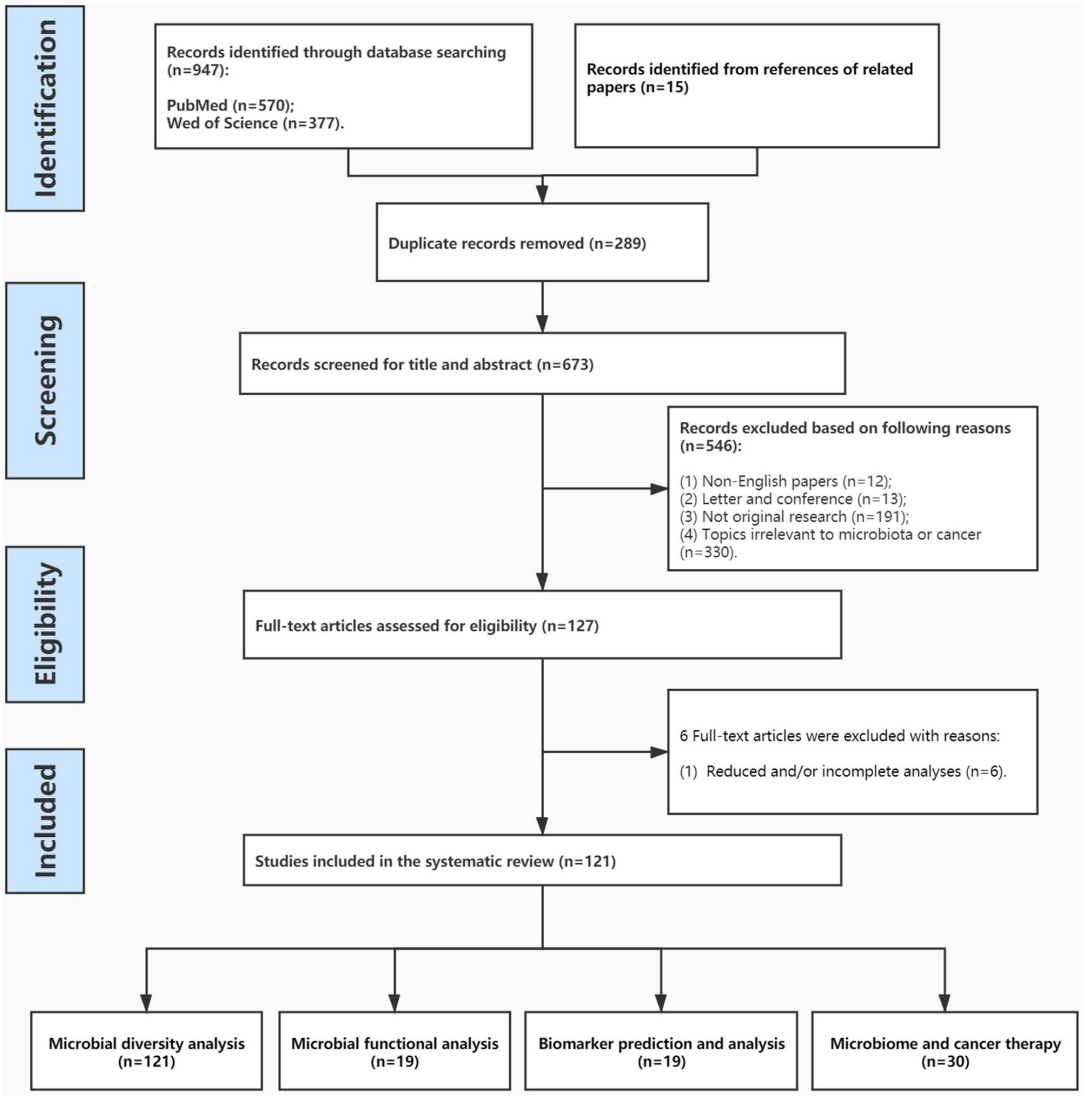
PRISMA graph detailing the search results.

The systematic review followed the PRISMA guidelines (Figure 1) (Stewart et al., 2015). Two investigators (LW and GY) independently conducted literature search using as combined keywords microbiome and cancer, security on PubMed (https://www.ncbi.nlm.nih.gov/pubmed/) and Web of Science (v. 5.35). The database search was run of all the published articles, all languages, from database inception until March 1, 2021. In both databases, the following search strategy was used: terms were searched as follows: Microbiome AND Cancer AND ^*^omics; Microbiome AND Cancer AND high-throughput; Microbiome AND Cancer AND genomics/metabolomics. ^*^omics was used in the search in order to identify longer forms. It is thought that these terms would be able to identify the majority of manuscripts within a narrow definition of microbiome and cancer and applied omics methodologies, though it remains likely that relevant sections might be embedded within methodology sections of particular projects and thus more challenging to identify.

### Study Selection and Data Synthesis

All studies reporting information on microbiome, cancer, high-throughput, and -omics were included. A total of 962 articles were identified and reviewed independently by two authors (LW and GY), and after all duplicates were removed, 673 articles were considered. After removing articles that were not in English, and those that had simply a mention of the words with no further expansion, 127 articles were considered. These articles are included in Table 1 for transparency and further reference. One hundred twenty-one articles (of the 127) devoted considerable amount of the manuscript to expand on those topics, while 6 articles had much reduced and/or incomplete analyses. Both of these latter categories were used in the current review. Any inconsistencies were resolved by consensus with a third author (ZK), while thematic groupings (Table 2) and analyses were reviewed by an additional author (IC). All outcomes were included, due to the wide range of use of the terminologies.

**Table 1:** Characteristics of included studies.

**Study**	**Subject of project**	**Type of cancer**	**High-throughput technology**	**Source of microbiota**
Zheng et al. (2011)	Human	Gastrointestinal cancers	Pyrosequencing	Formalin fixed and paraffin-embedded (FFPE) Biopsies
Zwielehner et al. (2011)	Human	Cancer^*^	16S rRNA	Fecal microbiota
Ganzenmueller et al. (2013)	Human	Verrucous skin tumors	Pyrosequencing	Virus
Gilbreath et al. (2013)	Human	Peritoneal tumors	16S rRNA	Tumor tissue
Hu et al. (2013a)	Human	Head and neck cancers	Pyrosequencing	Supragingival plaque
Hu et al. (2013b)	Human	Head and neck cancers	16S rRNA	Oral microbiota
Weir et al. (2013)	Human	Colorectal cancer	16S rRNA	Stool microbiome
Yamamoto et al. (2013)	Mice	Lymphoma	16S rRNA	Intestinal bacteria
Dejea et al. (2014)	Human	Colorectal cancers	16S rDNA	Colon tissues
Eun et al. (2014)	Human	Gastric cancer	16S rRNA	Gastric mucosal microbiota
Liang et al. (2014)	Mice	Colorectal cancer	16S rRNA	Gut microbiome
Montassier et al. (2014)	Human	Non-hodgkin's lymphoma	16S rRNA	Fecal microbiota
Wang et al. (2014)	Human	Leukemia	16S rRNA	Oral microbiota
Zackular et al. (2014)	Human	Colorectal cancer	16S rRNA	Gut microbiome
Hu et al. (2015)	Human	Gastric cancer	16S rDNA	Tongue coating microbiota
Pal et al. (2015)	Human	Metastatic renal cell carcinoma	16S rRNA	Stool bacteriomic
Montassier et al. (2015)	Human	Non-hodgkin's lymphoma	16S rRNA	Intestinal microbiome
Torres et al. (2015)	Human	Pancreatic cancer	16S rRNA	Salivary microbiome
Gao et al. (2015)	Human	Head and neck cancer	Pyrosequencing	Oral microbiota
Shelburne et al. (2015)	Human	Leukemia	16S rRNA; ITS sequencing	Oral and stool microbiome and mycobiome
Kasai et al. (2016)	Human	Colorectal carcinoma	16S rDNA	Gut microbiota
Galloway-Peña et al. (2016)	Human	Acute myeloid leukemia	16S rRNA	Gastrointestinal microbiome
Audirac-Chalifour et al. (2016)	Human	Cervical cancer	16S rDNA	Cervical microbiome
Montassier et al. (2016)	Human	Non-hodgkin lymphoma	16S rRNA	Gut microbiome
Walther-António et al. (2016)	Human	Endometrial cancer	16S rDNA	Uterine microbiome
Harris et al. (2016)	Human	Leukemia	16S rRNA	Gut microbiota
Thomas et al. (2016)	Human	Rectal carcinoma	16S rRNA	Fecal samples
Chng et al. (2016)	Human	Cholangiocarcinoma	16S rRNA	Tissue microbiome
Lu et al. (2016)	Human	Liver carcinoma	16S rRNA	Tongue coat microbiome
Moen et al. (2016)	Mice	Colorectal cancer	16S rRNA	Cecal microbiota
Lee et al. (2016)	Human	Lung cancer	16S rRNA	Microbiome in bronchoalveolar lavage
Salava et al. (2016)	Human	Melanomas	16S rRNA	Skin microbiome
Zhu et al. (2017)	Mice	Colorectal cancer	16S rRNA	Gut microbiota
Wolf et al. (2017)	Human	Oropharyngeal squamous cell carcinoma	16S rRNA	Salivary microbiome
Banerjee et al. (2017)	Human	Ovarian cancer	PathoChip microarray; Capture-next generation sequencing	FFPE samples
Frankel et al. (2017)	Human	Melanoma	Metagenomics	Gut microbiota
Gong et al. (2017)	Human	Laryngeal carcinoma	Pyrosequencing	Tumor tissues
Cavarretta et al. (2017)	Human	Prostate tumor	Pyrosequencing	Tumor tissues
Wang et al. (2017)	Mice	Colorectal cancer	16S rRNA	Gut microbiota
Yu T. et al. (2017)	Human	Colorectal cancer	16S rDNA	Gut microbiota
Yu J. et al. (2017)	Human	Colorectal cancer	Metagenomics	Fecal microbiome
Bučević Popović et al. (2018)	Human	Bladder cancer	16S rRNA	Urinary microbiome
Cong et al. (2018)	Human	Colorectal cancer	16S rRNA	Gut microbiota
Hakim et al. (2018)	Human	Acute lymphoblastic leukemia	16S rRNA	Gut microbiome
Herstad et al. (2018)	Dog	Colorectal epithelial tumors	16S rRNA; 16S rDNA	Fecal and mucosaassociated microbiota
Kwasniewski et al. (2018)	Human	Cervical cancer	16S rRNA	Cervical microbiota
Loke et al. (2018)	Human	Colorectal cancer	16S rRNA	Colon tissues
Meng et al. (2018)	Human	Breast cancer	16S rRNA	Breast tissue
Perera et al. (2018)	Human	Oral squamous cell carcinoma	16S rRNA	Oral fibroepithelial polyp
Stojanovska et al. (2018)	Mice	Colorectal cancer	16S rRNA	Fecal microbiota
Sun et al. (2018)	Human	Gastric cancer	16S rRNA	Oral microbiome
Wang et al. (2018)	Mice	Colorectal cancer	16S rRNA	Feces samples and intestinal tissues
Wu et al. (2018)	Human	Bladder cancer	16S rRNA	Urinary microbiota
Xue et al. (2018)	Rats	Breast cancer	16S rDNA	Intestinal flora
Yuan et al. (2018)	Mice	Colorectal cancer	16S rRNA	Gut microbiota
Zhang et al. (2018)	Mice	Colorectal cancer	16S rRNA	Gut microbiota
Fan et al. (2018)	Human	Pancreatic cancer	16S rRNA	Oral microbiome
Dai et al. (2018)	Human	Colorectal cancer	Metagenomics	Fecal microbiome
DeFilipp et al. (2018)	Human	Acute myeloid leukemia; myelodysplastic syndrome; non-hodgkin lymphoma	16S rRNA	Fecal microbiota
Gopalakrishnan et al. (2018)	Human	Melanoma	16S rRNA	Gut microbiome
Shah et al. (2018)	Human	Colorectal cancer	16S rRNA	Fecal microbiome
Routy et al. (2018)	Human	Epithelial tumors	Metagenomics	Gut microbiome
Matson et al. (2018)	Human	Metastatic melanoma	16S rRNA, metagenomics	Commensal microbiome
Ai et al. (2019)	Human	Colorectal cancer	Metagenomics	Gut microbiota
Alanee et al. (2019a)	Human	Prostate cancer	16S rRNA	Urinary and fecal microbiota
Alanee et al. (2019b)	Human	Suspected prostate cancer	16S rRNA	Urinary microbiome
Cho et al. (2019)	Human	Hepatocellular carcinoma	16S rDNA	Fasting serum samples
Cong et al. (2019)	Human	Colorectal cancer	16S rRNA	Intestinal microbiota
Diaz et al. (2019)	Human	Solid tumor	16S rRNA; ITS-1 DNA	Saliva
Han et al. (2019)	Human	Colorectal cancer	16S rRNA	Intestinal microorganisms
Ibrahim et al. (2019)	Mice	Colorectal cancer	16S rRNA	Gut microbiota
Jiang et al. (2019)	Human	Nasopharyngeal carcinoma	16S rDNA	Intestinal microbiota
Klein et al. (2019)	Human	Cervical cancer	16S rRNA	Cervical microbiome
Kong et al. (2019)	Human	Colorectal cancer	16S rRNA	Intestinal microbiota
Leung et al. (2019)	Human	Colorectal cancer	16S rRNA	Colonic microbiota
Liang et al. (2019)	Human	Gastric cancer	16S rRNA	Gut microbiota
Mai et al. (2019)	Human	Bladder cancer	16S rRNA	Urine bacteria
Ni et al. (2019)	Human	Primary hepatocellular carcinoma	16S rRNA	Gut microbiota
Qi et al. (2019)	Mice	Hepatocellular carcinoma	16S rDNA	Gut microbiota
Wang K. et al. (2019)	Human	Primary bronchogenic carcinoma	16S rDNA	Saliva and bronchoalveolar lavage fluid samples
Wang L. et al. (2019)	Human	Throat cancer	16S rRNA	Oral microbiota
Wongsurawat et al. (2019)	Human	Head and neck cancer	Metagenomics	Gut microbiome
Wu M. et al. (2019)	Mice	Colorectal cancer	16S rDNA; 18S rRNA	Gut microbes
Wu Y. et al. (2019)	Human	Colorectal cancer	16S rRNA	Colorectal cancer tissues
Xu et al. (2019)	Human	Gastric cancer	16S rDNA; 18S rRNA	Tongue coatings
Yang et al. (2019)	Human	Colorectal cancer	16S rRNA	Gut microbiota
Zhang B. et al. (2019)	Human	Multiple myeloma patients	16S rRNA	Fecal microbiota
Zhang L. et al. (2019)	Human	Primary liver cancer	16S rDNA	Gut microbes
Zheng et al. (2019)	Human	Gastric cancer	16S rDNA	Gastric juice or feces
Zhou et al. (2019)	Human	Ovarian carcinoma	16S rRNA	Ovarian cancer tissues
Feng et al. (2019)	Human	Prostate cancer	Metagenomic; Metatranscriptomics	Prostate microbiota
Peters et al. (2019)	Human	Melanoma	16S rRNA; metagenomics; metatranscriptome	Gut microbiome
Bian et al. (2020)	Mice	Colon cancer	16S rRNA	Gut microbiota
Clos-Garcia et al. (2020)	Human	Colorectal cancer	Metagenomics	Fecal metagenomics
Erawijantari et al. (2020)	Human	Gastric cancer	16S rRNA	Fecal microbiome
Ji et al. (2020)	Mice	Colorectal cancer	Metagenomics	Gut microbiota
Zhang Z. et al. (2020)	Mice	Colorectal cancer	16S rRNA	Gut bacteria
Zeng et al. (2020)	Human	Bladder cancer	16S rRNA	Urinary microbiome
Yu et al. (2020)	Mice	Colorectal cancer	16S rRNA	Gut microbiota
Xie et al. (2020)	Human	Cervical cancer	16S rDNA	Vaginal microbiota
Wei et al. (2020)	Human	Pancreatic cancer	16S rRNA	Oral microbiome
Wang W. J. et al. (2020)	Human	Colorectal adenoma	16S rDNA	Intestinal microflora
Wang, Q. et al. (2020)	Human	Colorectal cancer	16S rRNA	Gut mucosal microbiome
Sun et al. (2020)	Human	Pancreatic cancer	16S rDNA	Oral microbiome
Song and Gyarmati (2020)	Mice	Pediatric acute lymphocytic leukemia	16S rDNA	Gut microbiota
Shen et al. (2020)	Rats	Colorectal cancer	16S rDNA	Gut microbiota
Moskowitz et al. (2020)	Mice	Colorectal cancer	16S rRNA; metagenomics	Gut microbiota
Kim et al. (2020)	Human	Hepatocellular carcinoma	16S rRNA	Serum extracellular vesicles
Kang et al. (2020)	Human	Invasive cervical cancer	16S rRNA	Fecal microbiota
Hu et al. (2020)	Mice	Melanoma	16S rDNA	Intestinal microbiota
Chou et al. (2020)	Mice	Colorectal cancer	16S rRNA	Gut microbiome
Li et al. (2020)	Human	Liver cancer	16S rDNA	Oral microbiota
Liu M. et al. (2020)	Mice	Colon cancer	16S rRNA	Gut microbiota
Pan et al. (2020)	Rats	Esophageal tumorigenesis	16S rRNA	Gut microbiota
Heshiki et al. (2020)	Human	Lung cancer	Metagenomics	Gut microbiota
Nejman et al. (2020)	Human	Seven cancer types	16S rRNA; 16S rDNA	Tumor microbiome
Peled et al. (2020)	Human	Hematologic cancers	16S rRNA	Intestinal microbiota
Chung et al. (2021)	Human	Pancreatic cancer	16S rRNA	Oral, intestinal, and pancreatic bacterial microbiomes
Debesa-Tur (2021)	Human	Colorectal cancer	Metagenomics	Ffpe tissue
Jiang and Fan (2021)	Mice	Breast cancer	16S rDNA	Intestinal microbiota
Baruch et al. (2021)	Human	Melanoma	16S rRNA	Gut microbiota

* *Seventeen subjects receiving ambulant chemotherapy with antimicrobial therapy*.

**Table 2:** Thematic groupings of included articles.

**Groups/Thematic**	**Technology**	**Year**	**Number**	**Total number**	**Relationship to cancer**
	16S rDNA	2021	1	23	
		2020	6		
		2019	8		
		2018	2		
		2017	1		
		2016	3		
		2015	1		
		2014	1		
	16S rRNA	2021	2	82	
		2020	15		
		2019	20		
		2018	19		
		2017	3		
Microbial diversity and composition		2016	9		The microbial dysbiosis may lead to tumor microenvironment
analysis		2015	4		disturbance and contributes to cancer development.
		2014	5		
		2013	4		
		2011	1		
	ITS	2015	1	2	
		2019	1		
	Metagenomics	2021	1	14	
		2020	4		
		2019	4		
		2018	3		
		2017	2		
	Meta-transcriptome	2019	2	2	
	Pyrosequencing	2017	2	6	
		2015	1		
		2013	2		
		2011	1		
	PathoChip Microarray	2017	1	1	
	Capture-next Generation Sequencing	2017	1	1	
	16S rDNA	2020	1	1	
	16S rRNA	2020	4	15	
		2019	3		
		2018	5		
Microbial functional analysis		2017	1		Tumor microenvironment may result in functional alterations of
		2016	1		local microbiome, such as pathways related to
		2015	1		lipopolysaccharide biosynthesis and peptidases.
	Metagenomics	2020	2	5	
		2019	1		
		2018	2		
	16S rDNA	2020	1	5	
		2019	3		
Biomarker prediction and analysis		2016	1		Comparison of microbiome in healthy and tumoral samples
	16S rRNA	2020	1	13	using high-throughput technologies provides biomarker
		2019	3		candidates for prediction of cancer progression and mortality
		2018	3		such as γ-proteobacteria, *Adlercreutzia*.
		2017	1		
		2016	4		
		2014	1		
	Metagenomics	2020	1	2	
		2017	1		
	16S rDNA	2020	1	4	
		2019	2		
		2017	1		
	16S rRNA	2021	1	25	
		2020	3		
		2019	6		
Microbiome and cancer therapy		2018	7		Specific microbial species may interfere with tumor
		2017	2		progression or serve as predictive marker for cancer therapy.
		2016	3		
		2015	1		
		2014	1		
		2011	1		
	Metagenomics	2020	1	2	
		2017	1		

## Results

The manuscripts identified in this review (*n* = 121) followed four loosely defined thematic groups: (a) the methods used in measuring diversity (*n* = 121, i.e., all of the manuscripts used in this systematic review contained an element of measuring microbiome diversity), (b) the microbial functional analyses (*n* = 19), (c) the biomarker predictions (*n* = 19), and (d) microbiome in relation to cancer therapy (*n* = 30). They will be presented subsequently in this order, reflecting the scientific continuum, moving from the characterization and acquisition of knowledge, to the interpretation and finally toward clinical implementation. It becomes clear that both the number of technologies applied as well as the number of publications are increasing consistently, especially in the last few years, as can also be evidenced by the information on Table 2, and Table 1, with some studies deploying more than one methods in parallel. The most frequently used high-throughput technologies include in relative order of frequency amplicon sequencing, metagenomics, meta-transcriptomics, proteomics, and metabolomics. All of the above are accompanied with references, sometimes extensive, on continuously advancing bioinformatics analytical methods.

### Methods Used in Measuring Diversity and Composition

Diversity characterizing of the microbiome nowadays depends largely on cultivation-independent molecular technologies (Su et al., 2012) due to the unculturable property of the majority of microbes consisting the microbiome (Stewart, 2012; Browne et al., 2016). The sequential development of tools [including PCR-denaturing gradient gel electrophoresis (DGGE) (Scanlan and Marchesi, 2008; Zhang et al., 2010), fluorescence *in situ* hybridization (FISH) (Fredricks et al., 2005), quantitative dot blot hybridization, restriction fragment length polymorphism (RFLP) (Laguerre et al., 1994), terminal restriction fragment length polymorphisms (T-RFLP) (Wang et al., 2009), clone library (Bik et al., 2010; Rehman et al., 2011), and gene chip (Luo et al., 2020)] and the emergence of high-throughput sequencing technologies [16S/18S rRNA/rDNA gene sequence analysis (Fredricks et al., 2005; Scanlan and Marchesi, 2008; Rehman et al., 2011), high-throughput pyrosequencing (Rehman et al., 2011), metagenomics, meta-transcriptomics, and single-cell genomics (Lasken, 2007; Ishoey et al., 2008)] have broadened the perception of microbial diversity and evolutionary relationships of microbiota (Pace, 1997).

Initially, the sequence-based methods for analyzing microbiota relied on the first-generation sequencing technology developed by Sanger et al. (1977), which allowed culture-independent investigations (Morgan et al., 2017). However, these fingerprinting methods did not provide taxonomic information directly and were hard to detect rare or low-abundance taxa (Morgan et al., 2017). Subsequently, ribosomal RNA (rRNA) gene sequences in the conserved regions have been utilized to define and distinguish specific microbial species or populations from mixed organisms (Pace et al., 1986; Yarza et al., 2014) or to explore the bacterial diversity (Hugenholtz et al., 1998; Bik et al., 2010). Bik et al. determined the composition of oral bacterial diversity of 10 healthy individuals by constructing clone libraries from the amplified 16S rRNA gene, which was a comprehensive and high-resolution analysis of healthy human oral bacterial diversity in 2010 (Bik et al., 2010). Furthermore, combinations of two or more methods were utilized in an effort to avoid certain bias and discrepancies (Su et al., 2012), for example, deploying DGGE and ITS sequencing for analyzing the fungal diversity and richness in healthy human gut (Scanlan and Marchesi, 2008).

Responding to the high-throughput needs, the Biolog system also developed and provided phenotype microarrays specifically designed for microbiome analysis and ecological studies as a complement for traditional genomic, transcriptomic, and proteomic analyses, allowing users to conduct more targeted studies (Shea et al., 2012). PathoChip Microarray and Capture-next Generation Sequencing were also adopted to screen known pathogenic microbiomes including viruses, helminths, protozoa, fungi, and bacteria in ovarian cancer samples for investigating specific insertion sites of microbiome into the host genome, and that provided a solid association of microbiota with the ovarian cancer (Banerjee et al., 2017). Although microarray was a powerful tool to identify microbial species, only containing the known species of microbiota largely limited its application (Ehrenreich, 2006). The next-generation high-throughput sequencing avoided the system bias from the construction process of plasmid cloning library due to direct sequencing of the genome fragments (Pérez-Losada et al., 2018). The advantages of (eventual) low cost, high flux, good repeatability, and high accuracy provided a technological advantage and made it possible to profile the diversity of human gut microbiome comprehensively and to prevail in microbial ecology research (Liu Y.-X. et al., 2020).

Amplicon sequencing (Luo et al., 2020) is the most diffusely used method in microbiome analysis, as it is applicable to almost all sample types, provides vital insights into the microbial structural community, and helps to investigate the intricate and unsolved association between host and microbiome (Lynch and Pedersen, 2016). The main marker genes for amplicon sequencing include 16S rDNA for prokaryotes (Janda and Abbott, 2007) and 18S rDNA and ITS for eukaryotes (Shelburne et al., 2015; Dong et al., 2017; Diaz et al., 2019), among which 16S rDNA amplicon sequencing is currently the most commonly used method for detecting bacteria communities (Dejea et al., 2014; Hu et al., 2015; Audirac-Chalifour et al., 2016; Kasai et al., 2016; Montassier et al., 2016; Walther-António et al., 2016; Daniel et al., 2017; Herstad et al., 2018; Kwasniewski et al., 2018; Xue et al., 2018; Yuan et al., 2018; Cho et al., 2019; Leung et al., 2019; Qi et al., 2019; Zheng et al., 2019; Li et al., 2020; Sun et al., 2020; Wang W. J. et al., 2020; Zhang H. et al., 2020; Chung et al., 2021; Jiang and Fan, 2021). Some of the reasons for its wide adoptions are its ability to be used for low-biomass samples (Janda and Abbott, 2007) or for specimens contaminated with host DNA (Quince et al., 2017). Nonetheless, it does also have certain disadvantages, such as the biases and systematic errors induced during sampling, DNA extraction, library preparing, and sequencing (Hugerth and Andersson, 2017), environmental contaminations, or sample cross-talk (Edgar, 2016), potentially confusing primer sequences and limited genus-level resolution (Liu Y.-X. et al., 2020). In addition, the sensitivity to specific primers and selection of PCR cycle number may result in potential false-positive or false-negative results in downstream analysis (Liu Y.-X. et al., 2020). For analyzing the amplicon sequencing data, advanced specialized bioinformatic algorithms and pipelines were updated and adopted addressing biases, offering a better data quality, higher sensitivity, and higher specificity (Prodan et al., 2020). Collectively, operating taxonomic unit (OTU) clustering and amplicon sequence variant (ASV) analysis were two approaches for clustering and analyzing sequencing data based on either sequence identity (a threshold at 97%) or exact sequences with a statistical confidence (Zhai et al., 2020). Operating taxonomic units were normally used for evaluating the alpha-diversity of a microbial community (Hugerth and Andersson, 2017) by clustering similar sequences into a consensus sequence so as to filter and reduce noises or systematic errors in pipelines such as UPARSE (Edgar, 2010, 2013), MOTHUR (Schloss et al., 2009), or QIIME (Caporaso et al., 2010), whereas the ASVs showed great advantages when dealing with complicated samples or diminishing confounding factors that interfere with classification or analysis, especially its good performance on sensitivity and accuracy for big biomass (Caruso et al., 2019) in pipelines such as DADA2 (Callahan et al., 2016). Amplicon sequence variants have been proven to exhibit better sensitivity and specificity and distinguish microbial communities than OTUs (Callahan et al., 2016), even reaching species level or more (Callahan et al., 2017).

In recent years, metagenomics and meta-transcriptome are the two most rapidly advancing “omics” technologies (Aw and Fukuda, 2015), as they can monitor strain-level changes in microbiome and analyze potential functional activities of the gut microbiome in patients with cancer (Quince et al., 2017). For example, Yu J. et al. (2017) and Coker et al. (2019) revealed several gut species significantly associated with colorectal cancer (CRC) by metagenomics; in the oral squamous cell carcinoma, Yost et al. (2018) pointed out that *Fusobacteria, Selenomonas* spp., *Capnocytophaga* spp., and members of the genera *Dialister* and *Johnsonella* were significantly more active. Thus, high-throughput sequencing technologies have enabled the collection of comprehensive information on the gut microbiome and begun to reveal the correlation between microbiome and tumor (Zeller et al., 2014; Feng et al., 2015; Thomas et al., 2019; Yachida et al., 2019). While amplicon sequencing is a commonly used methodology for characterizing the microbiome due its lower cost, metagenomics and meta-transcriptome are more frequently applied to complex environmental samples.

Advantages and limitations of major high-throughput technologies are shown in Table 3. The integration of such multi-omic methodologies can provide further insights into cancer research (Liu Y.-X. et al., 2020). For example, Peters et al. (2019) characterized the gut microbiome for melanoma patients by 16S rRNA gene and shotgun metagenome sequencing and pointed out that the clustering of patients based on 16S microbiome composition was slightly more predictive of progression-free survival than clusters based on shotgun microbiome composition; on the other hand, species-level classification was much higher in the shotgun data, permitting researchers to identify more response-associated species than with 16S data alone.

**Table 3:** Advantages and limitations of major high-throughput technologies.

**Technologies**	**Advantages**	**Limitations**
•Amplicons (16S/18S /ITS)	•High-throughput, low-cost rapid detection •Flexibility to target one or more variable regions •Longer sequence reads and more accurate analysis •Identification with very low abundance	•Bias caused by PCR amplification, sequencing errors and chimeric sequences •Low repeatability and low quantification •Vulnerability to host genome interference
Pyrosequencing	•Rapid and accurate analysis of short DNA sequences with a high throughput capacity	•Limited read lengths
Metagenome	•More information •Functional Analysis •Identification of microbiota to species or strain level	•More expensive •Time-consuming •Host-generated contamination
Meta-transcriptome	•Detection of active microorganisms in the environment, active transcripts, and active functions •Comparison of differentially expressed genes and differential functional pathways in different environments	•The highest costs •The most complex sample preparation and analysis process •mRNA, and rRNA contamination of the host

### Microbial Functional Analysis

The application of high-throughput methodologies to the study of the human gut microbiome focuses not only on the microbiome composition but also on the functional analysis of the identified microbiome. Amplicon sequencing is a commonly used key tool for studying microbial communities as discussed above. The application of 16S rDNA (Dubin et al., 2019) or ITS rDNA using ASVs in DADA2 pipeline detected microbiome community in a high-resolution and high-accuracy way, which also helps identify the cross-kingdom dysbiosis and demonstrate the expansion and translocation pattern of pathogenic fungi during disease progression (Zhai et al., 2020). In addition, amplicon sequencing can also provide predictive functional analyses of microbial communities with quantifiable uncertainty if combined with advanced computational algorithms. PICRUSt was developed for predicting metagenomes according to amplicon sequencing data and reference genome databases (Langille et al., 2013). For example, QIIME and PICRUSt were utilized for diversity and compositional analysis and functional prediction after 16S rRNA sequencing, and it showed that proinflammatory pathways, such as lipopolysaccharide biosynthesis and peptidases, were enriched in the oral squamous cell carcinoma tissues and provided evidence for the inflammatory characteristic of bacteria related to cancer (Perera et al., 2018).

NGS-based methods provide the most common platform to explore metagenomic abundance of microbial community members at high genomic resolution (Quince et al., 2017). Specifically, shotgun metagenomics, i.e., the untargeted sequencing of all microbial genomes present in one sample, is a useful tool for quantifying microbiome and have been used to profile taxonomic composition and functional potential of microbial communities and to recover whole genome sequences (Quince et al., 2017). Databases that include combinations of manually annotated and computationally predicted proteins families, such as KEGG (Kanehisa et al., 2014; Erawijantari et al., 2020) or UniProt (UniProt Consortium., 2014), can be used for characterization of the functional potential of the microbiome. For instance, there were significantly lower catabolic pathway expression of local microbiota for responders to lung cancer therapy (Heshiki et al., 2020).

However, metagenomics still has limitations when it comes to profiling the active microbial community as measured by gene expression, a technological challenge addressed by meta-transcriptomics. Analysis of the meta-transcriptome, the mRNA of the microbiome, can reveal which organisms are active and which microbial genes are being expressed at the time of sampling under different conditions (Franzosa et al., 2014). For example, in prostate cancer, 10 *Pseudomonas* genes were found positively associated with eight host genes encoding small RNAs by such meta-transcriptome analysis (Feng et al., 2019). Furthermore, metagenomic functions related to progression-free survival were correlated with specific meta-transcriptomic expression patterns in melanoma patients (Peters et al., 2019). However, because of the short half-life of mRNA, such meta-transcriptome analyses represent a single time point of gene expression that may not necessarily reflect longer-term adaptations between the host and microbiota (Bikel et al., 2015). Therefore, integrating metagenomics and meta-transcriptomics enables the calculation of transcript/gene ratios, which represents an improved measure of gene transcriptional activation or repression (Bikel et al., 2015).

Additionally, different from the mRNA-based analyzing of meta-transcriptomics, meta-proteomics and metabolome are also new post-genomics high-throughput omics technologies for characterization of the whole protein component and all metabolites of microbiome at any given moment. They reveal the structural–functional diversity and dynamic changes at the protein level and metabolite level of microbes, which serves as potential biomarkers and enables an in-depth understanding of metabolic changes of microbial communities under diverse habitats (Johnson et al., 2016; Wilmanski et al., 2019; Dubey et al., 2020). The combination of metagenome and metabolome helped researchers to distinguish unique stage-specific phenotypes of the gut microbiota in CRC at the levels of species, genes, metabolic pathways, and metabolites (Yachida et al., 2019).

### Biomarker Prediction and Analysis

The number of microbes associated with the human body is estimated as at least 10 times that of human cells (Sender et al., 2016). Thus, initial investigations into microbes in cancer, such as the association between *H. pylori* and MALT lymphoma (Stolte, 1992), mainly focused on discovery, culture, and identification when they first emerged (Gilbert et al., 2016) using established and well-validated methodologies. Moreover, the characterization of microbiome in tumor remains challenging due to the low biomass of microbiota and methodological limitations (Nejman et al., 2020). Most microbiota was broadly considered as unculturable because of its tremendous genetic and biochemical diversity and the difficulties to mimic the natural living conditions in the laboratory (Stewart, 2012; Browne et al., 2016). High-throughput omics analyses are no longer limited to just detecting a few strains, but can detect microbiome in different microbiome niches in various cancer types (Nejman et al., 2020), thus providing a more comprehensive picture of the microbiome in relation to tumor development.

For example, such an analysis was performed in 1,526 samples from seven different types of solid tumors by applying a combination of methods including electron microscopy, H&E staining, immunohistochemistry (IHC), 16S rRNA FISH, qPCR, and culture *ex vivo*, coupled with high-throughput 16S rDNA sequencing (Nejman et al., 2020). This validated distinct microbial distributions in different tumor types and even across different subtypes of the same tumor type, which was also associated with bacterial prevalence and metabolic functions (Nejman et al., 2020). Accordingly, well-defined microbiome constituents can serve as a potential screen for early-stage cancer (Zackular et al., 2014) or a biomarker for prediction of cancer progression (Li et al., 2020). Re-analysis of raw 16S rRNA gene sequence data sets from nine separate studies in conjunction with a detailed meta-analysis and machine learning identified a composite microbial biomarker for diagnosing CRC consistent across studies (Shah et al., 2018). However, the low taxonomical and functional resolution of 16S rRNA sequencing limited the interpretation of the results beyond the accurate reach of species level (Shah et al., 2018). Multi-cohort metagenomic profiling studies highlighted and validated the potential of fecal metagenomic biomarkers for early non-invasive diagnosis of CRC even in different populations with distinct intestinal microbial community (Yu J. et al., 2017; Dai et al., 2018). The dynamic changes of microbial composition, gene abundance, and metabolites in gut microflora during the progression of CRC revealed by metagenomic and metabolomic analysis in a large cohort indicated microbial and metabolic shifts in the very early stages of CRC, which may contribute to a routine etiological diagnosis in the future (Yachida et al., 2019).

### Microbiome and Cancer Therapy

Besides the correlation of microbiome and cancer development, the same suite of methodologies is starting to be applied in order to characterize the therapeutic sensitivity or resistance to the treatment(s) of cancer(s). They can also contribute to discovering specific microbiota that influence the curative effects. For example, to examine the potential relationship between altered intestinal flora, CRC recurrence, and chemoresistance, investigators performed pyrophosphate sequencing and found that *Fusobacterium nucleatum* enriched in the CRC recurrent group promoted CRC chemoresistance *via* activating the cancer autophagy pathway (Yu T. et al., 2017).

16S rRNA sequencing, metagenomics, and metabolomics have been employed widely to reveal changes of intestinal or tissue microbiome in cancer patients treated with chemotherapy (Montassier et al., 2014, 2015, 2016; Wang et al., 2017; Hakim et al., 2018; Diaz et al., 2019), immune checkpoint inhibitors (Frankel et al., 2017), or surgery (Cong et al., 2018; Kong et al., 2019), and helped to predict the patient outcomes of cancer treatment. To name a few such examples, in a study based on high-depth sequencing results of 16S rRNA of fecal microbiota from children undergoing chemotherapy for newly diagnosed acute lymphoblastic leukemia, researchers linked the relative abundance of Proteobacteria before chemotherapy to the development of febrile neutropenia and found that domination of Enterococcaceae or Streptococcaceae in gut microbiome during chemotherapy predicted infection in subsequent phases of chemotherapy (Hakim et al., 2018). Moreover, immune checkpoint inhibitors targeting the programmed death 1 (PD-1) protein are important cancer therapeutics but have been reported failure for some patients probably because of dysbiosis in intestinal microbiome (Gopalakrishnan et al., 2018; Matson et al., 2018; Routy et al., 2018). In a research on patients with metastatic melanoma starting treatment with anti-PD-1 therapy, multiple high-throughput technologies, including 16S rRNA sequencing, metagenomic whole genome shotgun (WGS) sequencing, and whole exome sequencing, were utilized to reveal the association between diversity/relative abundance of Ruminococcaceae, Faecalibacterium, and Bacteroidales with the systemic and antitumor immune responses, which underlined the therapeutic potential of manipulating gut microbiome in patients with immune therapy (Gopalakrishnan et al., 2018). Additionally, a pilot study using 16S rRNA sequencing identified the changes of gut microbiota in post-surgery CRC patients and highlighted the key role of gut microbiota in the future care of surgical CRC patients (Cong et al., 2018). Shotgun metagenomic sequencing and metabolomic analysis based on capillary electrophoresis time-of-flight mass spectrometry revealed altered intestinal microbiome after gastrectomy and demonstrated its association with postoperative comorbidities (Erawijantari et al., 2020).

Application of sequencing-based high-throughput technologies enabled the scientists to observe the microbial dysbiosis at an integrated scale (Dong et al., 2018; Gopalakrishnan et al., 2018). In such a case, the microbiome and prognosis of allogeneic hematopoietic cell transplantation was investigated *via* 16S rRNA gene sequencing (Peled et al., 2020). The results demonstrated that higher microbial diversity during the transplantation period was associated with a reduced risk of death and increased overall survival, which can potentially be used as a biomarker to predict mortality in allogeneic hematopoietic cell transplantation patients (Peled et al., 2020). Notably, 16S rRNA gene and metagenomic sequencing of fecal samples in a phase I clinical trial suggested that performing fecal microbiota transplantation (FMT) treatment was associated with favorable changes in immune cell infiltration and gene expression profiles in the intestinal lamina propria and tumor microenvironment (Baruch et al., 2021). Overall, the integration of NGS methodologies with clinical analyses and treatment allowed one to observe the dynamic changes of gut microbiome and adjust the choice of treatment on tumor in time (Tanoue et al., 2019; Zheng et al., 2019; Erawijantari et al., 2020).

## Discussion

This manuscript is a systematic review of the application of high-throughput technologies to investigate both the microbiome and cancer. It is important to note that the studies identified in this review, using these high-throughput technologies, tend to focus more on characterizing the diversity of the microbiome as a whole and in cancer in particular. The large data volumes generated through -omics applications, e.g., genomics, metagenomics, and meta-transcriptomics, are frequently applied to the purposes of taxonomic composition profiling, functional annotation, and pathway enrichment analyses through computational approaches. This increasing application of omics enables also a better look into the dynamic changes and functional features of microbial communities under specific habitats, and for specific patient groups. As was evident by a number of identified publications, the latter analyses can also provide evidence for potential biomarkers or predictors for disease detection.

Lastly, a small number of publications demonstrated that avenues of applying such methodologies in the study of microbiome and cancer, in relation to therapy, have started to emerge. It is expected that the application of such high-throughput methodologies will continue, revealing the interrelationship between microbiome and cancer. The accrued understanding is anticipated to expand the potential of the microbiome as a prognostic indicator of cancer treatment, while high-throughput methodologies may also pave the way for new clinical interventions that alter composition and function of specific microbial communities in directions that might favor cancer therapeutic responsiveness.

Notwithstanding the above, the current review has some limitations. Specifically, the search included manuscripts that were identified in two online databases (PubMed and Web of Science) with parameters including year/language type/article type/keywords. This might have limited the breadth of the results. Additionally, pre-print databases, such as bioArxiv and F1000, were excluded as those manuscripts have not completed a peer-review process. In a field that is actively growing, such as the application of high-throughput technologies on microbiome and cancer, this strategy may lead to the omissions of the newest technologies currently under development. Furthermore, this review focuses on the application of these technologies without comparing potential integrating methodologies that may offer an additional layer of complexity.

## Conclusion

The emergence of high-throughput technologies enables in-depth studies on the relationship between microbiome and cancer. The ability to profile the microbiome as a whole, as well as the complex micro-ecosystems of the microbiome, enhances the possibility to use/measure specific microbial strata as predictive markers of cancer and eventually perhaps as a guide for precise treatments. However, these high-throughput methodologies produce high volumes of data and, as such, a downstream pressure for bioinformatics component able to ingest and interpret the results. Additionally, there still exist technical detection limits, especially with processing low-biomass samples.

Having said that, the majority of identified manuscripts in this review are still focusing their efforts on characterizing the microbiome and its relationship with cancer in detail. The many mechanisms by which the microbiome has the potential to modulate cancer development provide the possibility to target the microbiome for cancer prevention strategies. Additional clinically relevant data need to be generated, before microbiota-based strategies for cancer prevention can be envisioned and integrated into routine healthcare.

## Data Availability Statement

The original contributions presented in the study are included in the article/supplementary material, further inquiries can be directed to the corresponding author/s.

## Author Contributions

LW and GY conducted the systematic review and applied the eligibility selection criteria for the identified manuscripts. IC and ZK validated the selected manuscripts and arbitrated any queries. LW, GY, IC, and XL wrote the manuscript. NL, LD, and HW oversaw the process and provided critical input throughout. All authors were involved in the drafting of the manuscript.

## Author Disclaimer

Where authors are identified as personnel of the International Agency for Research on Cancer/WHO, the authors alone are responsible for the views expressed in this article and they do not necessarily represent the decisions, policy, or views of the International Agency for Research on Cancer/WHO.

## Conflict of Interest

The authors declare that the research was conducted in the absence of any commercial or financial relationships that could be construed as a potential conflict of interest.

## Publisher's Note

All claims expressed in this article are solely those of the authors and do not necessarily represent those of their affiliated organizations, or those of the publisher, the editors and the reviewers. Any product that may be evaluated in this article, or claim that may be made by its manufacturer, is not guaranteed or endorsed by the publisher.
